# Intellectual Property and Access to ART: Unwise Choice of Terminology

**DOI:** 10.1371/journal.pmed.0030509

**Published:** 2006-11-28

**Authors:** Richard Stallman

**Affiliations:** Free Software Foundation, Boston, Massachusetts, United States of America

The article “How Do Intellectual Property Law and International Trade Agreements Affect Access to Antiretroviral Therapy?” is very useful for its substance, but due to an unwise choice of terminology, it will tend to mislead the public in a way that the authors and editors probably are not aware of, which will promote the sorts of abuse that it seeks to criticize. This results from the use of the term “intellectual property.” This article uses the terms “intellectual property law” and “patent law” interchangeably, which is like using “Asia” and “India” synonymously. However, most readers will recognize the latter as loose use of language, so they will not really be led astray. Only a few will realize that identifying patents with “intellectual property law” is just as mistaken, so real confusion will result. I ask the editors of *PLoS Medicine*, and the readers and writers of articles, to be on guard against confusing use of the term “intellectual property”—which means, nearly all use of the term. See http://www.gnu.org/philosophy/not-ipr.xhtml for more explanation of the problems of this term.
